# Layered Clay–Graphene Oxide Nanohybrids for the Reinforcement and Fire-Retardant Properties of Polyurea Matrix

**DOI:** 10.3390/polym14010066

**Published:** 2021-12-24

**Authors:** Mădălina Ioana Necolau, Celina Maria Damian, Radu Claudiu Fierăscu, Anita-Laura Chiriac, George Mihail Vlăsceanu, Eugeniu Vasile, Horia Iovu

**Affiliations:** 1 Advanced Polymer Materials Group, University Politehnica of Bucharest, Gh. Polizu Street, 011061 Bucharest, Romania; madalina.necolau@upb.ro (M.I.N.); george.vlasceanu@upb.ro (G.M.V.); horia.iovu@upb.ro (H.I.); 2 National Institute for Research & Development in Chemistry and Petrochemistry-ICECHIM, 202 Spl. Independentei, 060021 Bucharest, Romania; fierascu.radu@icechim.ro (R.C.F.); anita-laura.radu@icechim.ro (A.-L.C.); 3 Department of Science and Engineering of Oxide Materials and Nanomaterials, University Politehnica of Bucharest, Splaiul Independentei 313, 060042 Bucharest, Romania; eugeniuvasile@yahoo.com; 4 Academy of Romanian Scientists, 54 Splaiul Independentei, 050094 Bucharest, Romania

**Keywords:** nanohybrids, polyurea nanocomposites, thermal properties

## Abstract

Nanostructures are more and more evolved through extensive research on their functionalities; thus, the aim of this study was to obtain layered clay–graphene oxide nanohybrids with application as reinforcing agents in polyurea nanocomposites with enhanced thermal–mechanical and fire-retardant properties. Montmorillonite (MMT) was combined with graphene oxide (GO) and amine functionalized graphene oxide (GOD) through a new cation exchange method; the complex nanostructures were analyzed through FTIR and XPS to assess ionic interactions between clay layers and GO sheets by C1s deconvolution and specific C sp3, respective/ly, C-O secondary peaks appearance. The thermal decomposition of nanohybrids showed a great influence of MMT layers in TGA, while the XRD patterns highlighted mutual MMT and GO sheets crystalline-structure disruption by the d (002) shift 2θ = 6.29° to lower values. Furthermore, the nanohybrids were embedded in the polyurea matrix, and the thermo-mechanical analysis gave information about the stiffness of MMT–GO nanocomposites, while GOD insertion within the MMT layers resulted in a 30 °C improvement in the Tg of hard domains, as shown in the DSC study. The micro CT analysis show good dispersion of inorganic structures within the polyurea, while the SEM fracture images revealed smooth surfaces. Cone calorimetry was used to evaluate fire-retardant properties through limiting the oxygen index, and MMT–GOD based nanocomposites showed a 35.4% value.

## 1. Introduction

Polyurea is a synthetic versatile elastomer used for high-strength coatings to protect different substrates from corrosion, temperature and weathering conditions. Polyureas, synthesized by rapid step-growth polymerization of isocyanates and polyether amines, have proved to be microphase-separated block copolymers with hard high-Tg domains represented by the rigid aromatic moieties embedded in relatively soft low-Tg matrix that come from the aliphatic polyamine [1,2,3].

**Scheme 1:**
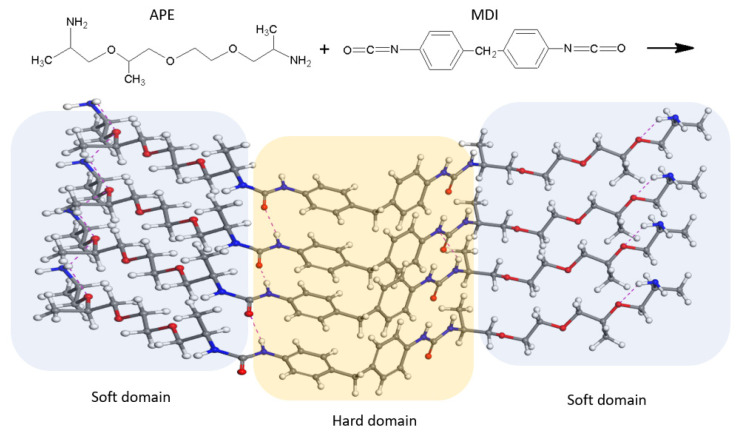
Schematic route for the polyurea matrix synthesis and the tribology of microphases.

The viscoelastic character of polyurea is a determining parameter and represents the topic of numerous studies in order to determine and understand their behavior and performances. A very important particularity for this material comes from the hard domains. Qiao et al. concluded in their study that the dynamic mechanical properties of polyurea can be enhanced by controlling the size, properties and distribution of hard domains [2]. The formation of numerous hydrogen bonds between urea polymeric chains, along with complementary interactions between adjacent aromatic structures from the hard domains endow this elastomer with superior mechanical resistance and rigidity (Scheme 1) [4].

Due to its rapid polymerization and moisture stability, polyurea offers a wide range of mechanical and chemical properties, from soft rubber to hard plastic, along with fire, abrasion, and corrosion resistance. Consequently, it has numerous applications in the coating industry, vehicles, pipelines, steel buildings and marine constructions [5].

One of the key findings of the study conducted by S.N. Raman was that the elastic modulus of polyurea was observed to be enhanced with the amplification of strain rates [6]. The high strain rates and the tensile behavior of this elastomeric material make it appropriate for strengthening structures and use as energy-absorbing materials in structural and ballistic systems.

Despite all of the abovementioned advantages, the applications for this polymeric material are quite limited due to the reduced fire-resistant capacity. The inherent flammability of polyurea emerges from the chemical structures and organic compositions and restricts the practical uses of this elastomer in the fields of electronics, construction and building, automobile and aerospace [7].

This shortcoming can be fixed by adding fire-resistant additives from the class of phosphorus compounds [8,9], but due to the high toxicity of resulting volatile compounds, researchers are looking for harmless alternatives. A feasible option would be the use of reinforcing agents, which have the ability to protect the material from heat by creating a physical barrier that can obstruct the permeation of free radicals, which are generated from combustion, within the material structure [10]. Recent advances in the development of functional nanocomposite materials indicate that nanofillers, such as carbon nanotubes (CNTs) [11], nanoclays [12] and graphene oxide (GO) [13], greatly improve the overall performance of polymers in a wide range of applications, among which fire-retardant effects are of great interest [14]. Layered silicates such as montmorillonite (MMT) bring stiffness to the nanocomposite, along with fire retardancy. At the same time, GO is known to be an important heat dissipater [15], although stacked GO nanosheets could suffer from low cross-plane thermal conductivity due to inter-sheet gaps, which is an obstacle for implementing effective heat dissipation. Thus, the paper offers two viable approaches: the synthesis of hybrid nanostructures between the two nanoreinforcing agents, together with the amine functionalization of GO.

This paper presents a series of new elastomeric nanocomposites based on the polyurea matrix and reinforced with different nanostructures based on montmorillonite and functionalized graphene oxide with enhanced mechanical and thermal properties. The materials were synthesized by sol–gel chemistry through a method that reduces the reactivity of the amino-terminated polymer. This increases the pot life of the mixture, with possible applications in protective coatings.

## 2. Materials and Methods

### 2.1. Materials

Jeffamine D230, which was used as a functionalization agent for the filler modification, was kindly supplied by Huntsman (Everberg, Belgium). Desmodur 44V20L (Covestro, Fribourg, Switzerland), a liquid dark brown polymeric diphenylmethane-4,4’-diisocyanate (MDI) which contains isomers and oligomers with NCO content between 30.5 and 32.5% wt., was used as an isocyanic component. O,O’-Bis (2-aminopropyl) polypropylene glycol-block-polyethylene glycol-block-polypropylene glycol (Jeffamine ED-900) (APE) with an amine index 1.80–2.25 and an approximate molecular weight of 900, purchased from Sigma Aldrich (Sigma-Aldrich Co., Steinheim, Germany), was used as amine component. Acetone (Aldrich, Steinheim, Germany) was used without further purification. Graphene oxide (GO) was synthesized, using a modified Hummers method described elsewhere [16], and fully characterized. Nanofil 116 montmorillonite (MMT), a natural layered silicate with a cation exchange capacity (CEC) of 116 mEq/100 g clay, was supplied by Southern Clay Products (Austin, TX, USA).

### 2.2. Methods

The synthesis of polyurea was carried out according to the approach proposed by Sanchez-Ferrer et al. [17] by using acetone as a solvent to reduce the reactivity of amine groups. A weight ratio of diisocyanate to amine of 1:2.7 at room temperature was considered. In two separate 10 mL flasks, the polyether amine (APE) (1.35 g in 3.5 mL of acetone) and the crosslinker (MDI) (0.5 in 1 mL of acetone) were dissolved. After 2 h, the contents of the two flasks were mixed in an ice bath and poured into the mold. The mixture was left for 24 h at room temperature for acetone evaporation and to make sure that the crosslinking process was completed.

**Scheme 2:**
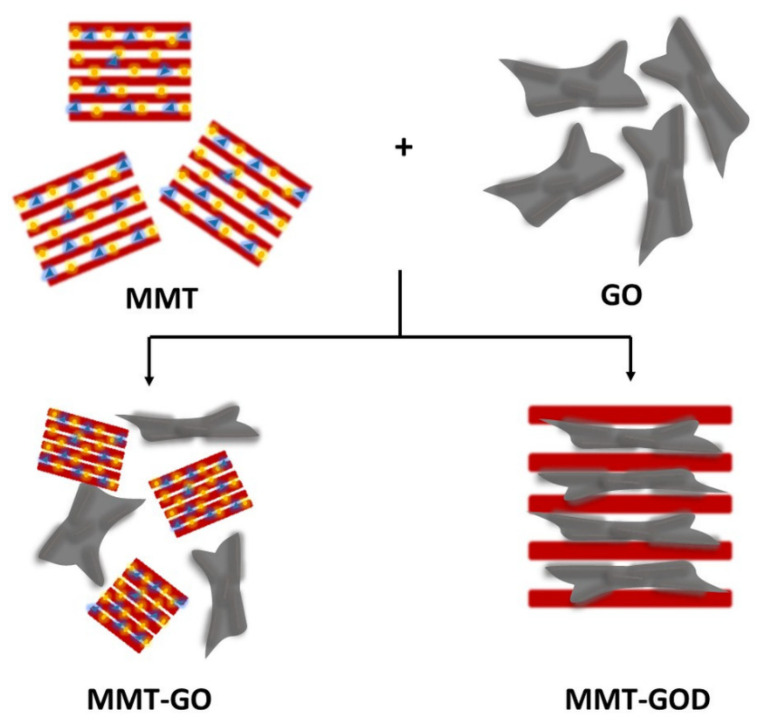
Synthesis route for MMT–GO and MMT–GOD nanohybrid structures.

GO functionalization with polyether diamine D230 was performed through a physical adsorption method by using the energy generated through ultrasonication. An amount of 1 mg/mL suspension of GO in ethanol was obtained, and then a 1% solution of D230 in ethanol was added. At the end, the suspension was washed with water, filtered and freeze-dried. MMT–GO nanohybrids were synthesized through a simple coupling reaction as follows: MMT was subjected to a swelling process in water at 80 °C for one hour, and then a 1 mg/mL GO suspension, namely HCl protonated GO functionalized with D230 suspension, was added. The structure of the synthesized hybrid nanocomposites is depicted in Scheme 2.

Polyurea/GO/MMT nanocomposites were obtained by dispersing 0.25 wt.% nanofiller in the diamine acetone solution, using a tip ultrasonicator for 1 h. After that, the resulting dispersion was mixed with the diisocyanate in acetone solution. The samples obtained through the dispersion of different types of reinforcing agents are presented in Table 1.

### 2.3. Characterization

The Fourier Transform Infrared Spectrometry (FTIR) was performed on Bruker VERTEX 70 equipment (Bruker, Billerica, MA, USA), using 32 scans in the 400–4000 cm^−1^ range, equipped with attenuated total reflection (ATR), using a Ge crystal.

X-ray Photoelectron Spectrometry (XPS) measurements were performed on a K-Alpha spectrometer (Thermo Scientific, East Grinstead, UK) with a monochromatic Al Kα source (1486.6 eV) and working in a vacuum base pressure of 2 × 10^−9^ mbar. Charging effects were compensated by a flood gun, and binding energy was calibrated by placing the C1s peak at 284.8 eV as internal standard. Deconvolution of C1s peaks was performed after Shirley background substraction. The pass energy for the survey spectra was 200 eV, and it was 20 eV for the high-resolution spectra.

The evaluation of the phase composition of the samples was performed by using X-ray diffraction (XRD) analyses (Rigaku Corp., Tokyo, Japan), with a Rigaku SmartLab equipment, operated at 45 kV and 200 mA, CuKα radiation (1.54059 Å), parallel beam configuration (2θ/θ scan mode).

Dynamic Mechanical Analysis (DMA) curves were registered on a TRITEC 2000 B device produced by Triton Technology, Ltd. Now Metler Toledo (Greifensee, Switzerland). The samples were analyzed in single cantilever bending mode. The samples were subjected to 1 Hz force and heated in a temperature range from −80 to 0 °C, with a heating rate of 4 °C/min.

Differential scanning calorimetry (DSC) analyses were performed on Netzsch 204 F1 Phoenix equipment (Selb, Germany) by heating the samples in aluminum crucibles from room temperature to 300 °C, in a nitrogen flow rate of 20 mL/min, at a heating rate of 10 °C/min.

Thermogravimetric analysis (TGA) was performed on TA Instruments Q500 equipment (Bellingham, WA, USA) as follows: a sample amount (about 3 mg) was heated in the temperature range of 20–800 °C, with a heating rate of 10 °C/min, in air and nitrogen atmosphere, using a platinum crucible.

Scanning electron microscopy (SEM) images were recorded on Quanta Inspect F (FEI, Hillsboro, OR, USA) equipped with a field-emission electron beam gun with a resolution of 1.2 nm and an energy dispersive X-ray spectrometer with a resolution of 30 kV. For optimum contrast, the surface was covered with a very thin gold conductor layer.

For micro-CT analysis, one composite specimen (approximately 1.5 × 1 × 1 mm) was analyzed with a Bruker microCT 1172 high-resolution micro-computer tomography scanner (Bruker, Kontich, Belgium), without further treatment. The sample was scanned with no filter, at a source voltage of 50 kV and a current intensity set for 200 µA, respectively. Overall, the scanned datasets were acquired during 180° rotations of the sample, with a rotation step of 0.3° and frame exposure of 1100 ms. Each slice was averaged from 5 successive acquisitions. Image pixel size—explicitly, the minimum resolution of the scanned dataset—was fixed at 1.5 µm, while the resolution of the projections was 4904 × 3280. In order to lessen the miscalculations, MMT–GO size distribution was performed on the majority of the available volume. Raw data reconstructions were performed in Bruker NRecon software. Reconstructed tomogram was depicted in Bruker CTVox, while quantitative determinations were performed in Bruker CTAn software.

Limiting oxygen index test (LOI) was performed by using a Stanton Redcroft FTA flammability instrument unit (Epsom, England), according to ISO 4589-2:2017 (E) standard. The downward flame propagation tests were standard. Upward flame propagation tests were conducted on vertical samples ignited at the bottom. Samples tested in flowing environments were 1 cm wide by 15 cm long

Mechanical tests were performed by using a universal mechanical tester (Instron, Model 3382, Norwood, MA, USA) at relative humidity of 45–50% and a speed of 1 mm/min. The size of the samples was approximatively 10 × 1 cm. A minimum of three specimens were tested for each polyurea samples, and the average values are reported. Tensile tests were performed by following the European Standard, EN ISO 527-3.

## 3. Results and Discussions

### 3.1. Nanostructures Characterization

**Figure 1:**
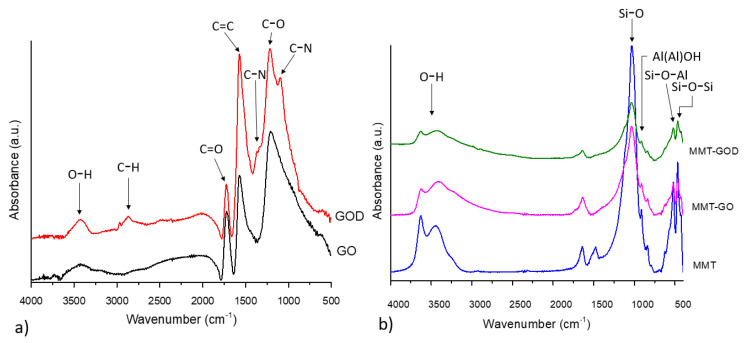
FTIR spectrum for: (**a**) GO nanomaterials and (**b**) MMT–GO based nanohybrid structures.

FTIR analysis is a valuable method to assess the structural modifications from the nanohybrids structures after functionalization; thus, the characteristic features for all the reinforcing agents were observed in the spectra depicted in Figure 1. All the MMT nanostructures exhibit distinctive peaks around 3630 cm^−1^ (Si-OH stretching vibration) [18], 3449 cm^−1^ (OH stretching vibrations), 1033 cm^−1^ (assigned to Si-O stretching), 913 cm^−1^ for Al(Al)OH bending [19], 913 cm^−1^ corresponding to Si-O-Si [20] and Si-O-Al and Si-O-Si at 520 respective 460 cm^−1^ stretching vibrations.

Regarding the graphene oxide spectrum, the presence of functional groups on its surface is supported by several peaks, including 3423 cm^−1^ for OH groups, 1716 cm^−1^ assigned to C=O bond, 1572 cm^−1^ from C=C bond and 1212 cm^−1^ corresponding to C-O. In the case of amine-functionalized graphene oxide (GOD), the peak that appeared around 2873 cm^−1^, confirms the presence of C-H stretching vibrations from the amine backbone. Other characteristic signals which support the success of the functionalization reaction can be found at 1218 and 1096 cm^−1^, referring to the C–O and C–N stretching vibrations of the amide group [21].

In the spectrum of MMT–GO and MMT–GOD nanohybrids, almost all the characteristic peaks of GO and MMT functional groups can be found, except for the absorption peak at 1723 cm^−1^, which is no longer present. The intensity of the OH absorption peaks at 3400–3600 cm^−1^ decreases as a consequence of the formation of hydrogen bonds between the graphene oxide nanosheet and clay layers [22]. All of these findings denote the success of the functionalization reaction between the two nanostructures.

**Figure 2:**
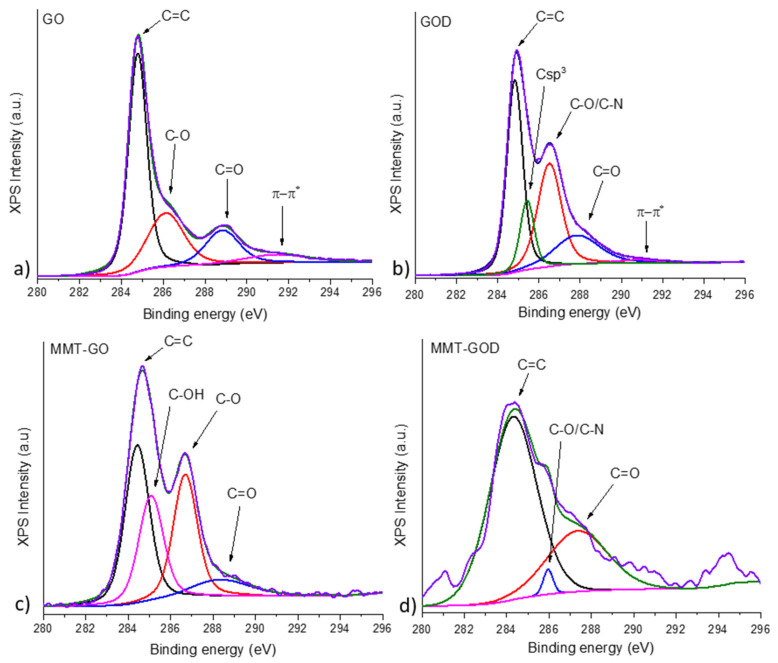
C1s high-resolution spectra for (**a**) GO, (**b**) GOD, (**c**) MMT–GO and (**d**) MMT–GOD.

X-ray photoelectron spectrometry is well-known to be among the most powerful tools to determine the structures of carbon-based materials; thus, the C1s level was deconvoluted into secondary peaks to assess structural modification of nanohybrids through functionalization. The high-resolution C1s spectrum registered for GO (Figure 2a) typically exhibits the following bands: C=C (at 284.78 eV), C-O-C (at 286.11 eV) and O-C=O (at 288.82 eV) and π–π* electronic transitions (at 291.43 eV). Meanwhile, in the case of GOD (Figure 2b), although similar components are found, the peak corresponding to C-O-C (at 286.51 eV) is observed to overlap with the one corresponding to the C-N species. When MMT- and GO-based nanohybrids are analyzed, there are several differences: in the case of MMT–GO (Figure 2c), the peak assigned to C- is more significantly related to the other secondary peaks, and this is an indicator of the presence of GO at the MMT surface [23]; meanwhile, for MMT–GOD (Figure 2d), besides the lower amount of carbon content (shown in Table 2), it can be seen that the C-O/C-N secondary peak is less significant, proving that the cationic exchange process occurred between MMT and GOD. Thus, most of the GOD molecules are located now within the MMT layers.

When looking at the surface elemental composition extracted from the survey XPS spectra (Table 2), it can be observed that the C1s percent is an indicator of surface modification. In comparison with GO, when functionalization was performed on GOD, the value of the C1s content is decreased, due to the presence of amine groups at the surface. Although the MMT structure shows adventitious carbon content [24], for MMT–GO nanohybrids, a 14.87 at.% C suggests that a part of the GO exists on the MMT surface. However, in the case of MMT–GOD, the clay layers were intercalated with GOD, which manages to penetrate within the MMT layers due to the higher compatibility offered by GO functionalization with D230; this hypothesis is sustained by the reduced C1s content at the surface compared with the MMT–GO nanohybrids.

**Figure 3:**
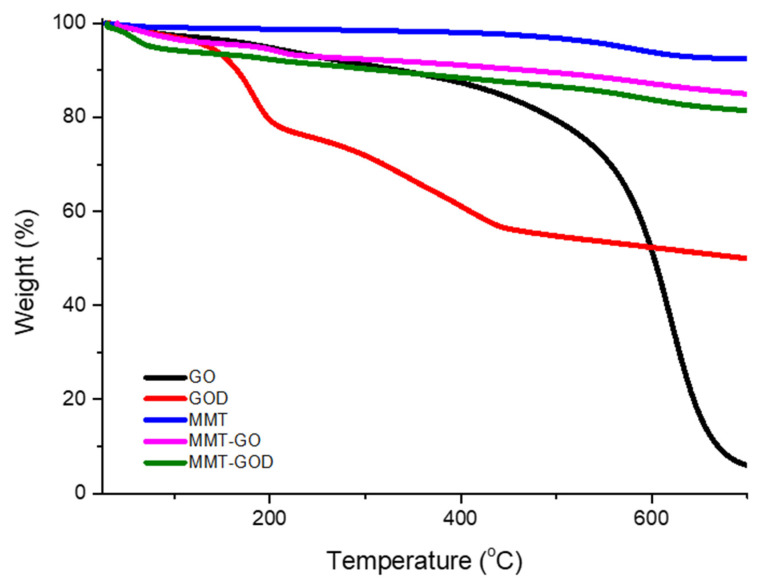
Thermal-degradation profile of nanohybrid structures.

The thermal degradation of nanoreinforcing agents through TGA revealed an efficient quantification for the functionalization stages starting with GO and MMT as raw materials: for the clay, we noticed its high thermal stability with a single-step degradation; meanwhile, for GO, the thermograms show a rapid degradation, which begins at 330 °C, caused by the decomposition of its surface oxygenated moieties, leading to a lower char amount. The TGA curve for GOD presents a different trend, due to the newly attached functional groups, leading also to an increase in the char content compared with neat GO (Table 3). In the case of nanohybrid structures, the thermal behavior of GO and GOD layers was masked by the good thermal stability of MMT. Their curves show similar shapes, as can be observed from Figure 3.

**Figure 4:**
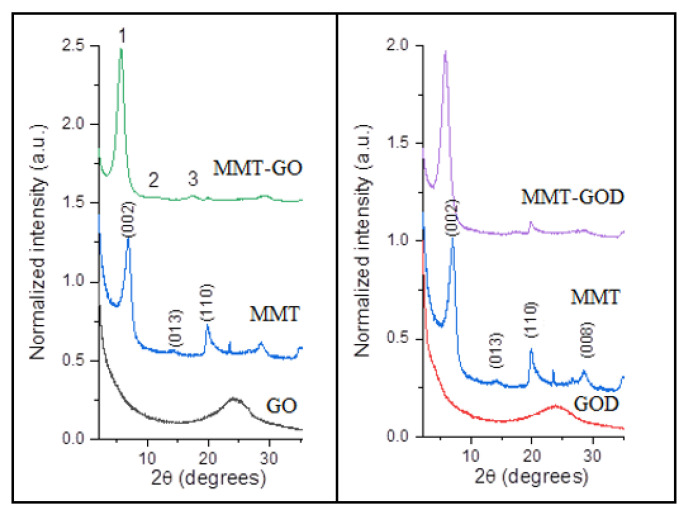
X-ray diffraction patterns for GO, GOD, MMT, MMT–GO and MMT–GOD.

For a better understanding of the modification process regarding the displacement of the sheets within nanohybrid formation, XRD analysis was performed on the GO, GOD, MMT and resulting MMT–GO and MMT–GOD structures (Figure 4). In the Bragg equation (2d sinθ = nλ), if 2θ is lower, the spacing of the nanosheets is larger.

For MMT nanohybrid structures, all the characteristic signals of MMT are present in the X-ray diffraction patterns; however, there are two peaks that need to be considered. It can be seen that there is a shift in the case of the first-order d-spacing peak d (001) reflection that is characteristic for MMT from 2θ of 6.29° to a lower 2θ of 5.74° for MMT–GO and 6.07° for MMT–GOD. When nanohybrids were analyzed, we noted that the characteristic peak remained and only slightly shifted a to lower angle; in addition, its shape became broader with decreased intensity, indicating that the effects between MMT and GO did not change the original layered structure of MMT. Moreover, the shift indicates an increase in the interlayer distance within the crystalline structure of the clay lamellae when functionalization with graphene oxide was realized [25,26]. This is an indicator that the GO was successfully intercalated between MMT platelets [23].

The diffraction peak at 2θ = 26.43° with an inter-planar distance of 3.6 nm in the spectrum of graphene oxide indicates exfoliated graphene nanosheets separated in the form of a few-layered structure [27]. The decrease of 2θ at 23.88° for GOD suggests a higher distance between the graphene sheets after the amine functionalization reaction [28,29].

The characteristic 2θ peaks at 24.14° and 23.88° for the GO and GOD structure, respectively, are not translated into the nanohybrid diffractograms, accordingly showing that these structures are altered by the interaction with MMT. However, it can be seen that MMT–GO and MMT–GOD nanohybrids are keeping, with lower intensity and slightly shifted, the characteristic 002, 110 and 013 reflection features from the layered clay structure. This is an indicator that MMT layers are connected with GO sheets through weak interactions.

### 3.2. Composite Materials Characterization

**Figure 5:**
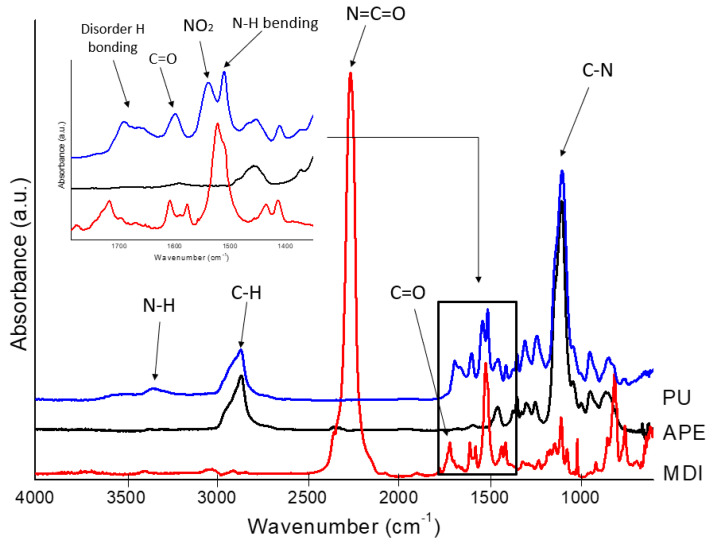
FTIR spectra for the polyurea matrix and raw materials used for its synthesis.

The chemical structure of the polyurea was characterized and confirmed by using FTIR analysis, and the spectra are shown in Figure 5.

The isocyanate crosslinker showed a specific strong band at 2266 cm^−1^, correlated with the N=C=O asymmetric stretch, which is consumed during the reaction of polyurea formation, as it can be observed from the PU spectrum. The signal at 1521 cm^−1^ was ascribed to the NO_2_ antisymmetric vibration, and the peak at 1718 cm^−1^ corresponds with the carbonyl C=O group.

The functional groups in the final product were identified as follows: the urea functional group was confirmed according to the peaks at 1600 cm^−1^ (C=O stretching vibration), 1511 cm^−1^ (N-H bending vibration), 2867 cm^−1^ (C-H stretching vibrations) and 3330 cm^−1^ (characteristic stretching vibration for N-H in the urea functional group) [30,31].

The peaks at 1662 cm^−1^ indicates the hydrogen bonding formed by one C=O of the urea groups with two nearby N-H. This interaction is conventionally called “disordered” bonding. The strong absorption peak at 1100 cm^−1^ is attributed to the isocyanate C-N stretch, while the C-O stretching vibration is represented by the presence of the signals at 1304 cm^−1^, 1235 cm^−1^ and 945 cm^−1^. The spectra also show a sharp peak at 1540 cm^−1^, revealing the presence of nitro compounds NO_2_ [32].

**Figure 6:**
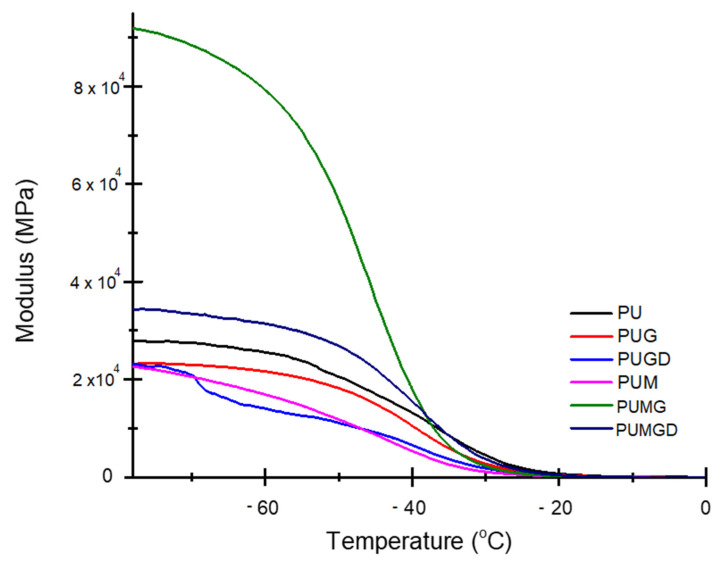
Storage modulus, ε’, for the polyurea based nanocomposites.

The polyurea matrix exhibits a combination of elastic and viscous properties that can be explained in standard engineering terms, using DMA methods. It can be seen from Figure 6, that ε exhibited a quasilinear stability with the temperature in the glassy state and dropped rapidly due to the changes which occur as a result of intramolecular friction when passing to rubbery state [33].

By analyzing the storage modulus curves, it can be noticed that the PUM, PUG and PUGD samples have slightly lower values in the glassy state for ε’ when compared with the PU matrix, due to the presence of the reinforcing agent that interfered and disrupted the hard segment content that is responsible for the mechanical properties [34], inducing rigidity throughout its inorganic structure. When PUMGD nanocomposites are analyzed, only a slight increase is observed as compared with neat matrix.

In the case of the PUMG samples, the storage modulus curves indicate a superior elastic response by increased values of ε’ when compared with the PU matrix within the glassy state; this result may be a consequence of the synergistic interaction of the nanostructures with the polymeric matrix, proving that a good dispersion and strong interfacial interaction between the nanofiller and the polymeric matrix occurred. At the same time, the functional groups from the GO surface can interact with the amide groups from the polyurea molecules, playing a key role in the formation of nanoscale rigid domains, thus leading to the formation of an ideal crystalline phase/amorphous phase ratio in the final composite materials [35,36].

The DSC data revealed that the samples subjected to analysis had two characteristic Tg temperatures (Table 4). The first corresponds to the soft areas and provides information on the elasticity of the samples, and the second temperature corresponds to the hard areas and is responsible for the stiffness of the synthesized material. The two values indicate the micro-phase separation morphology of the material [37,38,39]. As shown in Table 4, the best elastic behavior is represented by the PUG sample, due to the insertion of large flexible GO sheets; meanwhile, in terms of rigidity, the PUGD sample is noted to have the highest Tg of hard domains. In an overall look through the Tg values of nanocomposite samples, one can observe that the values for soft domains are particularly influenced by the structure of the reinforcing agents. Thus, a low Tg for PUG could have come from large GO sheets, but, at the same time, a second hypothesis can be that the reactants were hindered to reach active sites by aggregates; therefore, elastic behavior is enhanced by excess. However, when functionalized GO is used as a reinforcing agent, the value of soft Tg is higher, due to the fact that lower dimensions of GOD aggregates or functionalization of the GO can possibly increase the crosslinking density through participation in the polyurea formation of the amine groups from the D230 molecule. In the case of MMT nanostructures, the first pattern mentioned above is also observed. Possible agglomeration of MMT nanoclay lead to decreased values for Tg. However, the addition of GO sheets to MMT clay modified the nanostructures dimensions and, thus, their distribution within the polyurea network. The soft regions of PUMG and PUMGD have similar values for Tg. The main difference between these two samples resides in the hard region, where PUMGD shows a higher value for Tg.

**Figure 7:**
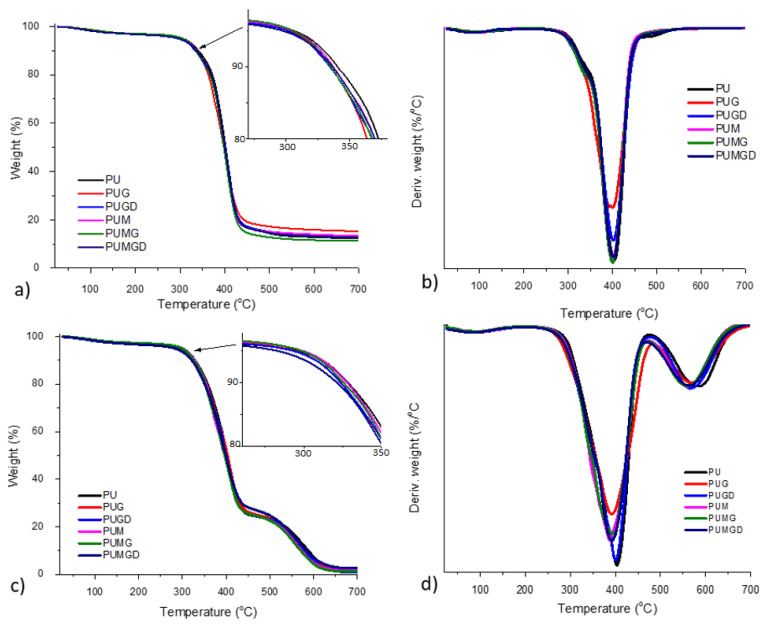
The TG (**a**) and DTG (**b**) diagrams of PU and PU nanocomposites under nitrogen atmosphere. The TG (**c**) and DTG (**d**) diagrams of PU and PU nanocomposites under air atmosphere.

The thermal properties were assessed by using thermogravimetric analysis (TGA) to obtain the thermal stability of the newly synthesized materials in inert (N2) and oxidative (Air) atmospheres (Figure 7 and Table 5). Upon exceeding a temperature threshold, a thermo-oxidative process occurred between 290 and 330 °C. The result of this process, which started with breaking the chemical bonds in the PU matrix chain, was the release of some volatile products, thus leading to the decrease of the initial mass of the analyzed sample.

Regarding this aspect, in a degradation study realized by Awad and Wilkie, carbon dioxide was found to be preponderant in the first stage of degradation, while, in the second stage, isocyanate end groups (i.e., HNCO and CH3NCO, respectively) and hydrocarbon fragments CxHy were predominant [39]. In this study, this oxidative process was not significantly influenced by the presence of the reinforcing agent, probably due to its low amount. However, an increase in thermal stability was observed in the case of PUMGD, and this outcome can be connected to the hypothesis of amine involvement in the PU-formation reaction. In this case, a higher crosslinking density can hinder the bond scission propagation throughout the sample network. At the same time, the lower Td5% values obtained for the nanocomposites reinforced with nanohybrid structures can be explained by the fact that a large number of centers that are susceptible to inducing accelerated decomposition were introduced by the aggregates formed between the clay layers and GO sheets. The presence of MMT does not influence the decomposition process, because the inorganic material is highly thermally stable.

The thermal degradation of PU and its nanocomposites was evaluated by TGA, and the results are shown in Figure 7 and Table 5, respectively. As it can be seen, under a nitrogen atmosphere (Figure 7a,b), the PU and its nanocomposites present two degradation stages at 280–330 °C and 330–453 °C. The first step of thermal degradation occurs due to the degradation of the hard segments of urea groups which have lower thermal stability. The second stage is assigned to the soft segments which are generated by the long polyether amine chains and possess higher thermal stability [40]. However, in the case of TGA analysis under air atmosphere (Figure 7c,d), one more decomposition peak occurs in the temperature range of 480–600 °C, as compared with that under nitrogen atmosphere. This step can be attributed to the thermo-oxidative degradation of the polymeric residue [41].

**Figure 8:**
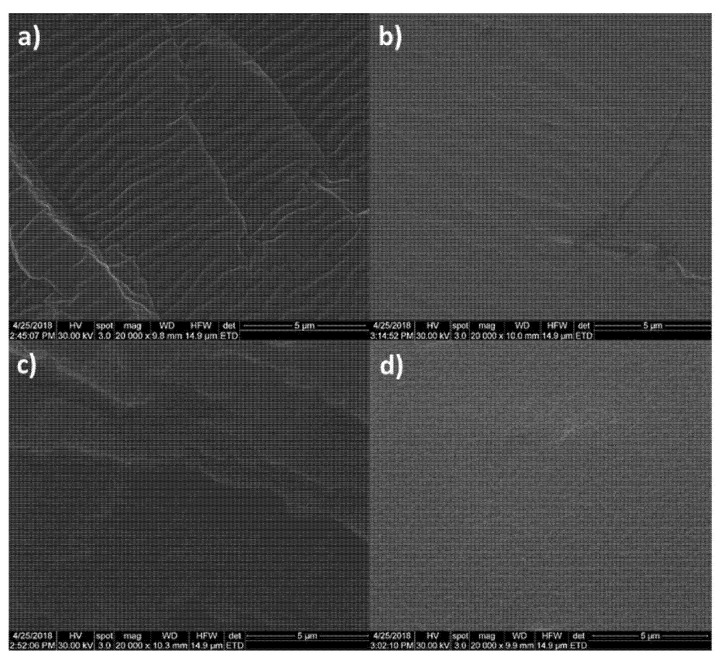
Morphological characterization by SEM for: (**a**) the polyurea matrix and (**b**) the nanocomposites PUG, (**c**) PUM and (**d**) PUMG.

The fracture characteristic morphology shown by the SEM images (Figure 8) revealed that the introduction of the nanoreinforcing agent disrupted the PU network arrangement. This disruption can be better observed in the case of PUMG (Figure 8d), where a smooth surface was obtained. At the same time, surface compatibility between nanoreinforcing agent and polyurea matrix can be ascribed to the roughness of the fracture surface. Thus, one can observe an efficient embedding of the reinforcing agent within the PU matrix. However, PUG and PUM (Figure 8b,c) still have some wiggles, which can indicate GO and MMT pull-out at fracture.

**Figure 9:**
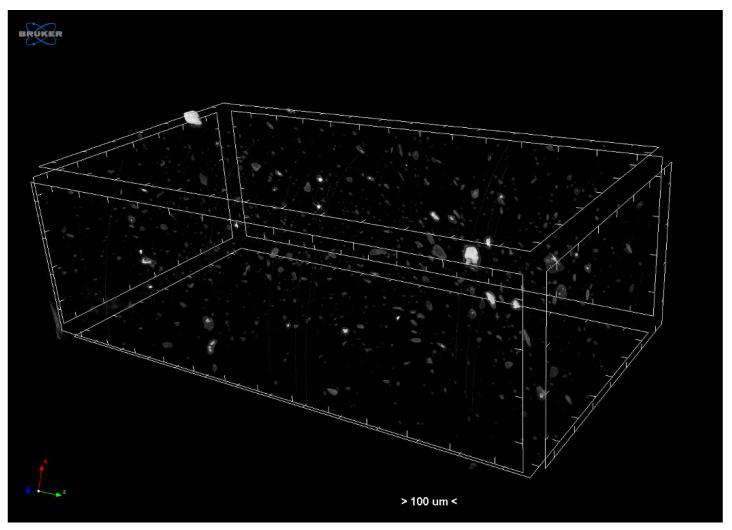
CT scan for MMT–GO polyurea sample.

Micro-computed tomography (micro-CT) was used for the visualization and quantification of the nanoreinforcing agent into the polyurea matrix. For the PUMGD composite specimen (Figure 9), one rectangular volume of interest (VOI) dataset was extracted. The VOI was constrained in terms of volume and height to a mass at a maximum of 99% of the scanned specimen. In CTAn, the VOI was processed through a common procedure consisting of thresholding, which was performed to clear-cut the sample’s inorganic phase from the polymer matrix; despeckling, which was used for the removal of residual scanning artefacts; and 3D analysis, which was conducted to quantify the specific surface (total clay surface/volume ratio) of MMT–GOD agglomerates, total volume of the filler and structure thickness, a granulometric measure of the clay distribution within the matrix. Reconstructed tomograms are grayscale images, with a gray palette covering 0 (black) to 255 (white). For organic matrices reinforced with inorganic nanoparticles, thresholding was carried out with ease, as the difference in X-ray attenuation coefficient is high; hence, the clays are depicted in light gray tones. Post-thresholding, the quantitative 3D analysis was carried out.

Some studies state that polyurea has an ideal morphology for mechanical properties [42]. Mott and et al. pointed out in their study that the response of polyurea to mechanical perturbation is strongly rate-dependent and associated with substantial energy loss [43].

The mechanical properties of polyurea are also influenced by hydrogen bonds. High mechanical toughness is a result of the extensive intermolecular hydrogen bonding in polyurea hard domains [38]. Many studies have demonstrated the influence and behavior of these bonds during stress and deformation. In the case of nanocomposites, the molecular weights of polyurea, the dispersion of nanostructure and the hydrogen bonds formed with the polyurea matrix are the three decisive factors for the mechanical strength. Qian et al. reported that the low addition of graphene oxide (0.2 wt%) substantially increased the tensile strength and elongation of polyurea, but the overloading of graphene oxide affected the polymerization of polyurea [35].

The stress–strain result shows that the polyurea samples have different behaviors, ranging from a highly deformable soft rubber to a rigid, brittle material when subjected to mechanical stress, as shown in Table 6. In comparison with the neat PU sample, the most significant increase in the Young’s modulus values can be noticed for the PUMG sample. The reduced values of the Young’s modulus of the PUMGD can be related to the formation of complex networks between the nanostructures used as reinforcing agents and the matrix, leading to the plasticization given by the unreacted amine. In the case of PUM, structural compatibility issues could lead to agglomerated MMT layers, which could hinder the mobility of polyurea reactants. The stiffest nanocomposite according to the values for the Young’s modulus is the PUMG sample. This aspect confirms that the reinforcing agent used in this case has a more agglomerated structure embedded within the polymeric matrix, contributing to the mechanical properties of the final material, in accordance with DMA storage modulus curves.

As overall mechanical properties, the MMT- and MMT–GOD-reinforcing agents interfere in the structural organization of the polymeric matrix and lower the mechanical properties. Meanwhile, for the GO-reinforced polyurea sample, the higher value for the tensile stress comes from the ability of the graphene oxide nanosheet to provide flexibility to the system by unfolding when nanocomposites are subjected to load [44].

The flame-retardancy behavior of polyurea samples was assessed by using limiting-oxygen-index (LOI) experiments, and the results are summarized in Table 7. According to the ASTMD 2863-97 standard, a material in which the LOI value is above 23 ± 2% is rated as flame retardant. Compared with the neat polyurea sample, a slight increase in the value of LOI can be seen in the case of nanocomposite samples reinforced with graphene and montmorillonite. This may be due to the formation of a barrier at the material surface that limits the oxygen permeability into the material and the evaporation of volatile compounds and prevents the heat transfer. The most significant values for LOI can be noticed in the case of PUMG and PUMGD samples for which GO and MMT may exhibit a synergistic effect towards the flame retardancy of the nanocomposites [44]. This statement is also supported by TGA results in which the value for the char residue is correlated with the LOI, which assesses high thermal stability. The literature data for PU-based materials showed that carbon dioxide is mainly released after decomposition [45].

## 4. Conclusions

Combined MMT and GO nanohybrid structures were synthesized through a novel approach based on cationic exchange between layered clay and amine-functionalized GO nanosheets in order to obtain a synergistic effect for both nanomaterials. The comprehensive structural characterization given by FTIR, XPS and XRD techniques showed the success of the concept. Thus, the intensity of OH characteristic bands from the FTIR spectra of MMT–GO and MMT–GOD nanohybrids was decreased, while sp3 species from XPS C1s secondary band deconvolution were no longer present in the MMT–GOD structure, giving a vision about the cation exchange mechanism which permits the insertion of amine-functionalized sheets within the MMT layers. These findings corroborated with the slight shift of the characteristic MMT signal from 2θ = 6.29°, confirming the synthesis of MMT–GOD nanohybrids. Polyurea nanocomposites, a class of versatile coating materials, were further synthesized to gain an advanced synergistic effect of MMT and GO in the nanohybrids. The thermal properties were analyzed, and the results from DSC showed, by an increased high domain Tg, that amine functional groups were embedded within the polyurea matrix. Furthermore, the LOI of PUMG and PUMGD nanocomposites was significantly improved, suggesting an efficient transfer of properties from the nanohybrids based on GO and MMT in terms of the flame-retardant properties. Future work is planned to consolidate the proposed nanohybrid synthesis mechanism, based on different types of modifier amines, as well as the flame-retardant and energy-dispersive effects of polyurea coatings on different substrates.

## Figures and Tables

**Table 1:** Abbreviations used for polyurea formulations prepared.

No.	Sample	Name
1	PU–APE	PU
2	PU–APE–GO	PUG
3	PU–APE–GOD	PUGD
4	PU–APE–MMT	PUM
5	PU–APE–MMT–GO	PUMG
6	PU–APE–MMT–GOD	PUMGD

**Table 2:** Surface elemental composition from XPS for the nanoreinforcing agents.

Atomic %	GO	GOD	MMT	MMT–GO	MMT–GOD
C1s	79.42	76.75	5.13	15.54	13.01
O1s	20.58	18.75	55.94	53.35	53.77
N1s	-	4.50	-	-	1.24
Si2p	-	-	23.57	20.43	21.20
Mg1s	-	-	2.74	3.33	2.76
Al2p	-	-	7.36	6.34	6.86
Ca2p	-	-	2.15	1.01	1.16
Na1S	-	-	3.11	-	-

**Table 3:** Thermal properties for the nanoreinforcing agents.

Sample	Thermal Decomposition Range			Char (%)	T_d3%_ (°C)
	Stage I	Stage II	Stage III		
GO	331–752 °C	-	-	4	125
GOD	96–238 °C	238–491 °C	-	46	102
MMT	350–714 °C	-	-	92	493
MMT–GO	45–149 °C	149–263 °C	263–898 °C	83	94
MMT–GOD	36–140 °C	140–245 °C	491–899 °C	85	60

**Table 4:** Glass-transition temperature values obtained by DSC analysis for polyurea and its nanocomposites.

Sample	Tg Soft (°C)	Tg Hard (°C)
PU	−30	32
PUG	−47	37
PUGD	−36	54
PUM	−39	40
PUMG	−32	44
PUMGD	−32	65

**Table 5:** TGA data for polyurea nanocomposites.

Sample	Air			Nitrogen		
	T_d5%_(°) ^1^	T_d10%_(°) ^2^	T_max_(°) ^3^	T_d5%_(°)	T_d10%_(°)	T_max_(°)
PU	296	329	406	304	340	404
PUG	296	325	391	304	345	400
PUGD	294	324	401	301	334	403
PUM	300	328	386	305	337	400
PUMG	299	326	395	305	335	400
PUMGD	283	321	392	297	337	399

^1^ T_d5%_ = decomposition temperature at 5% mass loss; ^2^ T_d10%_ = decomposition temperature at 10% mass loss; ^3^ T_max_ = temperature associated with the maximum degradation rate.

**Table 6:** Mechanical properties for the polyurea based nanocomposites.

Sample	E (MPa)	Tensile Stress (%)
PU	0.105	42.2
PUG	0.610	61.4
PUGD	0.555	47.3
PUM	0.268	44.5
PUMG	1.034	40.2
PUMGD	0.235	29.6

E—Young’s modulus calculated from tensile tests.

**Table 7:** Flammability tests for polyurea nanocomposite samples.

Sample	Limiting Oxygen Index (LOI) ^1^ (%)	Residual Mass ^2^ (%)—Air	Residual Mass ^2^ (%)—Nitrogen
PU	32.4	0.98	12.33
PUG	33.6	1.16	15.19
PUGD	33.8	0.92	13.22
PUM	33.8	1.98	13.31
PUMG	35.2	1.29	11.25
PUMGD	35.4	2.57	12.63

^1^ Data from flammability test; ^2^ data from thermogravimetric analysis.

## Data Availability

Not applicable.

## References

[1] Li T., Zhang C., Xie Z., Xu J., Guo B.-H. (2018). A multi-scale investigation on effects of hydrogen bonding on micro-structure and macro-properties in a polyurea. Polymer.

[2] Qiao J., Amirkhizi A.V., Schaaf K., Nemat-Nasser S., Wu G. (2011). Dynamic mechanical and ultrasonic properties of polyurea. Mech. Mater..

[3] Mattia J., Painter P. (2007). A Comparison of Hydrogen Bonding and Order in a Polyurethane and Poly(urethane−urea) and Their Blends with Poly(ethylene glycol). Macromolecules.

[4] Castagna A.M., Pangon A., Choi T., Dillon G.P., Runt J. (2012). The Role of Soft Segment Molecular Weight on Microphase Separation and Dynamics of Bulk Polymerized Polyureas. Macromolecules.

[5] Shim J., Mohr D. (2009). Using split Hopkinson pressure bars to perform large strain compression tests on polyurea at low, intermediate and high strain rates. Int. J. Impact Eng..

[6] Raman S., Ngo T., Lu J., Mendis P. (2013). Experimental investigation on the tensile behavior of polyurea at high strain rates. Mater. Des..

[7] He W., Song P., Yu B., Fang Z., Wang H. (2020). Flame retardant polymeric nanocomposites through the combination of nano-materials and conventional flame retardants. Prog. Mater. Sci..

[8] Awad W.H., Wilkie C.A. (2011). Further study on the flammability of polyurea: The effect of intumescent coating and additive flame retardants. Polym. Adv. Technol..

[9] Arunkumar T., Ramachandran S., Sebastian P.J., Vipin Raj C. (2015). Thermal and fire retardant behaviour of polyurea. Int. J. Appl. Eng. Res..

[10] Khobragade P.S., Hansora D.P., Naik J.B., Chatterjee A. (2016). Flame retarding performance of elastomeric nanocomposites: A review. Polym. Degrad. Stab..

[11] Emin ÇETİN M. (2022). Investigation of carbon nanotube reinforcement to polyurethane adhesive for improving impact per-formance of carbon fiber composite sandwich panels. Int. J. Adhes. Adhes..

[12] Elmergawy F.H., Nassif M.S., El-Borady O.M., Mabrouk M., El-Korashy D.I. (2021). Physical and mechanical evaluation of dental resin composite after modification with two different types of Montmorillonite nanoclay. J. Dent..

[13] Fadil Y., Thickett S.C., Agarwal V., Zetterlund P.B. (2021). Synthesis of graphene-based polymeric nanocomposites using emulsion techniques. Prog. Polym. Sci..

[14] Nizam Uddin M., Le L., Nair R., Asmatulu R. (2019). Effects of graphene oxide thin films and nanocomposite coatings on flame retardancy and thermal stability of aircraft composites: A comparative study. J. Eng. Mater. Technol. Trans ASME.

[15] Hong S., Yoo S.S., Yoo P.J. (2019). Binder-free heat dissipation films assembled with reduced graphene oxide and alumina nano-particles for simultaneous high in-plane and cross-plane thermal conductivities. J. Mater. Chem. C.

[16] Ciobotaru C.C., Damian C.M., Matei E., Iovu H. (2014). Covalent functionalization of graphene oxide with cisplatin. Mater Plast..

[17] Sánchez-Ferrer A., Rogez D., Martinoty P. (2010). Synthesis and Characterization of New Polyurea Elastomers by Sol/Gel Chemistry. Macromol. Chem. Phys..

[18] Arabkhani P., Asfaram A., Ateia M. (2020). Easy-to-prepare graphene oxide/sodium montmorillonite polymer nanocomposite with enhanced adsorption performance. J. Water Process. Eng..

[19] Kim D., Mittal G., Kim M., Kim S.H., Rhee K.Y. (2018). Surface modification of MMT and its effect on fatigue and fracture behavior of basalt/epoxy based composites in a seawater environment. Appl. Surf. Sci..

[20] Chen D., Chen J., Luan X., Ji H., Xia Z. (2011). Characterization of anion–cationic surfactants modified montmorillonite and its application for the removal of methyl orange. Chem. Eng. J..

[21] Nimita Jebaranjitham J., Mageshwari C., Saravanan R., Mu N. (2019). Fabrication of amine functionalized graphene oxide—AgNPs nanocomposite with improved dispersibility for reduction of 4-nitrophenol. Compos. Part B Eng..

[22] Zhou X., Hu B., Xiao W.Q., Yan L., Wang Z.J., Zhang J.J., Lin H.L., Bian J., Lu Y. (2017). Morphology and properties of shape memory thermoplastic polyurethane composites incorporating graphene-montmorillonite hybrids. J. Appl. Polym. Sci..

[23] Wang C., Ge X., Jiang Y. (2019). Synergistic effect of graphene oxide/montmorillonite-sodium carboxymethycellulose ternary mimic-nacre nanocomposites prepared via a facile evaporation and hot- pressing technique. Carbohydr. Polym..

[24] Li Y., Chen H., Wu J., He Q., Li Y., Yang W., Zhou Y. (2018). Preparation and characterization of APTES modified magnetic MMT capable of using as anisotropic nanoparticles. Appl. Surf. Sci..

[25] Stanly S., Jelmy E., Nair C., John H. (2019). Carbon dioxide adsorption studies on modified montmorillonite clay/reduced graphene oxide hybrids at low pressure. J. Environ. Chem. Eng..

[26] Ganjaee Sari M., Shamshiri M., Ramezanzadeh B. (2017). Fabricating an epoxy composite coating with enhanced corrosion re-sistance through impregnation of functionalized graphene oxide-co-montmorillonite Nanoplatelet. Corros Sci..

[27] Ali M.E. (2019). Preparation of graphene nanosheets by electrochemical exfoliation of a graphite-nanoclay composite electrode: Application for the adsorption of organic dyes. Colloids Surf. A Physicochem. Eng. Asp..

[28] Stobinski L., Lesiak-Orłowska B., Małolepszy A., Mazurkiewicz-Pawlicka M., Mierzwa B., Zemek J., Jiricek P., Bieloshapka I. (2014). Graphene oxide and reduced graphene oxide studied by the XRD, TEM and electron spectroscopy methods. J. Electron Spectrosc. Relat. Phenom..

[29] Yang S., Ren X., Zhao G., Shi W., Montavon G., Grambow B., Wang X. (2015). RETRACTED: Competitive sorption and selective sequence of Cu(II) and Ni(II) on montmorillonite: Batch, modeling, EPR and XAS studies. Geochim. Cosmochim. Acta.

[30] Ying Z., Wu C., Zhang C., Jiang S., Shi R., Cheng H., Zhang B., Li Y., Zhao F. (2017). Synthesis of polyureas with CO 2 as carbonyl building block and their high performances. J. CO2 Util..

[31] Arunkumar T., Ramachandran S. (2016). Surface coating and characterisation of polyurea for liquid storage. Int. J. Ambient. Energy.

[32] Jiang M., Liu Q., Zhang Q., Ye C., Zhou G. (2011). A series of furan-aromatic polyesters synthesized via direct esterification method based on renewable resources. J. Polym. Sci. Part A Polym. Chem..

[33] Holzworth K., Jia Z., Amirkhizi A., Qiao J., Nemat-Nasser S. (2013). Effect of isocyanate content on thermal and mechanical properties of polyurea. Polymer.

[34] Qian X., Song L., Tai Q., Hu Y., Yuen R.K. (2013). Graphite oxide/polyurea and graphene/polyurea nanocomposites: A comparative investigation on properties reinforcements and mechanism. Compos. Sci. Technol..

[35] Casalini R., Bogoslovov R., Qadri S., Roland C. (2012). Nanofiller reinforcement of elastomeric polyurea. Polymer.

[36] Cai D., Song M. (2014). High mechanical performance polyurea/organoclay nanocomposites. Compos. Sci. Technol..

[37] Fragiadakis D., Gamache R., Bogoslovov R., Roland C. (2010). Segmental dynamics of polyurea: Effect of stoichiometry. Polymer.

[38] Grujicic M., He T., Pandurangan B., Svingala F.R., Settles G.S., Hargather M.J. (2012). Experimental characterization and materi-al-model development for microphase-segregated polyurea: An overview. J. Mater. Eng. Perform..

[39] Awad W.H., Wilkie C.A. (2010). Investigation of the thermal degradation of polyurea: The effect of ammonium polyphosphate and expandable graphite. Polymer.

[40] Zhang T., Cai W., Chu F., Zhou F., Liang S., Ma C., Hu Y. (2019). Hydroxyapatite/polyurea nanocomposite: Preparation and multiple performance enhancements. Compos. Part A Appl. Sci. Manuf..

[41] Roland C.M., Twigg J.N., Vu Y., Mott P.H. (2007). High strain rate mechanical behavior of polyurea. Polymer.

[42] Mott P.H., Giller C.B., Fragiadakis D., Rosenberg D.A., Roland C.M. (2016). Deformation of polyurea: Where does the energy go?. Polymer.

[43] Wolk A., Rosenthal M., Weiß J., Voigt M., Wesendahl J.-N., Hartmann M., Grundmeier G., Wilhelm R., Meschut G., Tiemann M. (2018). Graphene oxide as flexibilizer for epoxy amine resins. Prog. Org. Coat..

[44] Thi N.H., Pham D.L., Hanh N.T., Oanh H.T., Yen Duong T.H., Nguyen T.N., Tuyen N.D., Phan D.L., Trinh H.T., Nguyen H.T. (2019). Influence of Organoclay on the Flame Re-tardancy and Thermal Insulation Property of Expandable Graphite/Polyurethane Foam. J. Chem..

[45] Giraud S., Bourbigot S., Rochery M., Vroman I., Tighzert L., Delobel R., Poutch F. (2005). Flame retarded polyurea with micro-encapsulated ammonium phosphate for textile coating. Polym. Degrad. Stab..

